# Landmark-Based Homing Navigation Using Omnidirectional Depth Information

**DOI:** 10.3390/s17081928

**Published:** 2017-08-22

**Authors:** Changmin Lee, Seung-Eun Yu, DaeEun Kim

**Affiliations:** School of Electrical and Electronic Engineering, Yonsei University, 50 Yonsei-ro, Seodaemun-gu, Seoul 03722, Korea; lcmin@yonsei.ac.kr (C.L.); catheun@gmail.com (S.-E.Y.)

**Keywords:** landmark navigation, homing navigation, distance sensor, distance-estimated landmark vector, landmark vector, arrangement order matching

## Abstract

A number of landmark-based navigation algorithms have been studied using feature extraction over the visual information. In this paper, we apply the distance information of the surrounding environment in a landmark navigation model. We mount a depth sensor on a mobile robot, in order to obtain omnidirectional distance information. The surrounding environment is represented as a circular form of landmark vectors, which forms a snapshot. The depth snapshots at the current position and the target position are compared to determine the homing direction, inspired by the snapshot model. Here, we suggest a holistic view of panoramic depth information for homing navigation where each sample point is taken as a landmark. The results are shown in a vector map of homing vectors. The performance of the suggested method is evaluated based on the angular errors and the homing success rate. Omnidirectional depth information about the surrounding environment can be a promising source of landmark homing navigation. We demonstrate the results that a holistic approach with omnidirectional depth information shows effective homing navigation.

## 1. Introduction

A number of methods have been developed for the challenging issue of autonomous navigation of mobile robots [1,2,3,4,5]. The methods vary widely, from landmark-based navigation [6,7], which is inspired by insect homing behavior, to complex algorithms that require intensive computation and continuous tracking of the information about the environment [1,8,9,10]. In homing navigation methods, the agent can assess an environmental feature obtained at the current position and can compare it to that at a target position. By comparing the feature information at two locations, the agent can determine which direction to move in to reach the target position. This concept has been proposed as the ‘snapshot model’ [11,12,13]. A snapshot taken at the current location is compared with a snapshot at the home location to obtain the direction from the current position to the home location. Various methods have been suggested for homing navigation, and among them, we focus on homing algorithms that are based on the snapshot model.

One snapshot method, the warping method [14,15], distorts the current snapshot image in order to best match the target snapshot image. The agent predicts a new image for every possible direction that it could move from the current location by warping the snapshot image. Each warped image is compared to the target image by searching for the one with the smallest discrepancy. The warped image with the smallest difference indicates the direction in which to move in order to reach the target location. The performance of the predictive warping method is largely affected by the characteristics of the environment and the existence of a reference compass; however, the method is simple and does not require any pre-processing of feature extraction in the snapshot image.

The Descent in Image Distance (DID) method extends the concept from the warping method. The DID method was introduced by Zeil et al. and was investigated from various perspectives [16,17]. While the one-dimensional warping method only searches for the minimum difference from the home image among the candidate images, the DID method monitors variations of the image difference between a pair of locations. The difference between snapshots decreases as the location becomes closer to the home point. By applying a gradient descent method, the navigation algorithm successfully finds the direction to the goal location [15,18].

One of the simplest homing navigation methods is the Average Landmark Vector (ALV) model [6]. At each location, the mobile robot takes a snapshot image, and each landmark in the view composes a landmark vector. Each landmark vector has a unit length with an angle as the corresponding angular position of the landmark. The ALV is computed by averaging all of the landmark vectors. The ALV computed at an arbitrary position is compared to that at the home location. By comparing only two averaged landmark vectors, the agent can determine the appropriate direction to the home location. Therefore, this navigation method has a small computing time and requires a small amount of memory. This method shows good homing navigation performance using a simple representation of the surrounding environment [19].

The Distance-Estimated Landmark Vector (DELV) model is another landmark-based homing navigation algorithm [20]. While the ALV model produces and compares two ALVs at different positions, the DELV method compares two sets of landmark vectors in order to determine the homing direction. These two navigation algorithms share similar concepts of landmark vectors, pointing to landmarks at a given position. However, while the landmark vectors in the ALV model have a unit length, DELV includes the length of the vector as the estimated distance to each landmark. Thus, the DELV method possesses more detailed information about the surrounding environment around the agent through the use of distance-estimated landmark vectors, and as a result, the agent estimates the current location with respect to the home location. In addition, the matching process of the landmark vectors in the DELV model does not require the reference compass information, while the ALV model necessarily does.

The DELV model was initially tested using vision sensors, so an additional distance estimation process is required for selected landmarks [21,22]. Based on the visual information, variations in the landmark position between snapshots obtained at a sequence of robot positions can lead to the computation of the landmark distance. However, by exploiting the depth sensor, the distance information to each landmark can be given along with the landmark selection. Extracting the landmark feature may be a painful procedure, since in some cases, the accurate identification of each landmark and the determination of what landmarks should be selected in a real environment are non-trivial problems. The homing performance with DELV or ALV becomes much worse in a real environment than in an artificial environment with clearcut landmarks.

The localization issue is a challenging issue, even in modern Simultaneous Localization And Mapping (SLAM) technology. Information about the environment can also be obtained from several types of sensors. The global localization map can be obtained with a global positioning system (GPS) and a laser-ranging sensor. The laser range sensor can also be tested for human robot interaction [5] or industrial applications [23]. In addition to the distance information about the environment, the Monte Carlo approach with an elevation map of objects or three-dimensional features commonly observed from the air and ground can be used to provide better localization information [24,25]. The landmark-based methods described above collect particular features called landmarks, and the snapshot model simplifies the navigation issue into the homing navigation. A mobile robot needs no localization map with the landmark-based method and thus the method efficiently determines the homing directions without identifying the current location. However, a high level of knowledge such as the environmental map cannot be obtained. In contrast, the SLAM method can utilize the localization map to guide a specific goal position with more computing time and memory.

Visual information has been most widely applied to navigation algorithms, since it can provide a lot of information about the position, brightness, color, and shape of objects. Many navigation algorithms are developed with feature extraction using vision [26,27,28], often with a complex filtering process [29]. Even simple landmark-based navigation methods, including the ALV model, have been applied to a mobile robot with vision sensors, and a feature extraction process to identify landmarks is also required [26]. In contrast, the DID method is a holistic approach for comparing a pair of snapshot images pixel by pixel to calculate the image distance [15,16,17]. It has no feature extraction process; however, computing the gradient of the image distance is needed to determine the homing direction.

The main contribution of this paper is in suggesting a holistic approach with the omnidirectional depth information including the background for homing navigation in a real environment, which is called H-DELV. It is a landmark-based navigation with distance information, but an effort of extracting landmark features can be omitted with the suggested approach. We assume that the environment roughly has an isotropic landmark distribution where the frequency of landmarks are independent of the viewing direction and the majority of landmarks are commonly observed irrespective of the view. We explore the efficient and robust landmark-based navigation of a mobile robot without prior knowledge of landmarks such as the colors or shapes of landmarks. We will show that one-dimensional panoramic distance information is sufficient to localize the agent in the environment to guide the homing performance. We suggest a holistic approach of reading landmarks using omnidirectional depth information for homing navigation in a real environment. In our experiments, we test the homing navigation based on landmark segmentation over the depth information, as well as that based on a holistic view of the depth information.

We analyze the effect of landmark vector strategies using data from various experiments, changing the weight of landmark vectors, using the reference compass or not, varying the number of landmark vectors, and adding the feature extraction or clustering process. Our paper seeks answers to the following questions: What are important factors that influence the homing performance in the snapshot model as a bio-inspired model? What kind of landmark vector strategies will be useful if the distance sensor readings are available? What strategies will be helpful in an environment assuming an isotropic landmark distribution? Which strategy, a holistic view of landmarks or a landmark distribution involved with the feature extraction, will be more effective in real environments? Is the robotic homing model similar to the model for insect navigation?

## 2. Methods

Initially, we explain the robotic configuration for homing navigation. From the depth sensor, omnidirectional depth information as the depth snapshot is obtained by following the snapshot model. How landmarks are extracted in the snapshot determines which landmark vector strategies can be used. We will introduce two new approaches based on the landmark distance vectors, C-DELV and H-DELV and compare those with the conventional DELV method. In this section, we will explain the three methods one by one.

### 2.1. Mobile Robot with a Depth Sensor

**Figure 1:**
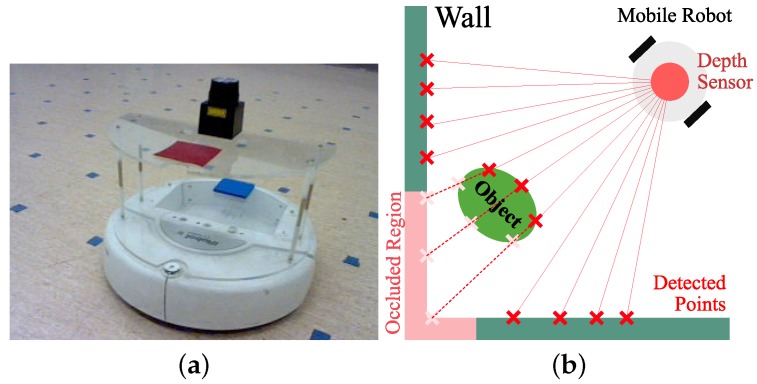
Mobile robot and sensor reading example (**a**) a mobile robot Roomba robot mounted with a laser sensor as the depth sensor; (**b**) an example of sensor readings with the laser sensor (red *x* marks show the distance readings for given angles).

For our navigation experiments, a mobile robot was used with a distance sensor as shown in Figure 1a. The mobile robot (Roomba, Create 4400, iRobot, Bedford, MA, USA) is a two-wheeled robot that can control the motor speed. A laptop computer processing the sensor readings from a laser sensor or vision sensor was connected to the robot via serial port for motor control. Depth information was obtained with a laser sensor device (URG-04LX-UG01, HOKUYO, Osaka, Japan)mounted on top of the Roomba with an acrylic support. The laser sensor can read distance information from the environment, which is then transmitted to a laptop computer on the robot. Figure 1b shows an example of distance sensor readings, where red *x* marks with an interval of angular resolution indicate detection points. In addition, one object makes some occlusion area on the wall which is marked in pink. The depth sensor has a distance accuracy of 5 mm (within a range of 60–1000 mm) or 30 mm (within a range of 1000–4095 mm) and an angular resolution of about 0.36${}^{\circ }$. We collect omnidirectional distance information for one snapshot at a given location, that is, a set of distances for the whole range of angles.

The computer processes a landmark-based algorithm and transfers the appropriate motor actions to the robot. For a panoramic image (depth map) of the surrounding environment, the mobile robot takes two snapshots at a given location using the laser sensor, since the laser sensor has a limited viewing angle, $240^{\circ }$ for a snapshot. The $360^{\circ }$ omni-directional depth information was obtained by overlapping the two snapshots. The mobile robot Roomba runs with its own battery pack and it can sustain for a period of 1.5 h (on a hard floor with no payloads or attachments) when the robot constantly moves. The laptop computer has a separate battery pack that also supplies power (about 2.5 Watts) for the depth sensor with USB connection. The laptop computer uses MATLAB software (MATLAB, 2014b, MathWorks, Natick, MA, USA) to run the control algorithm and consumes 7–11 watts. With the batteries, the whole system can operate for about an hour.

To check the performance in terms of the homing direction, we used a regular grid of points on the floor as well as additional random positions to see the performance, where small markers were placed for easy detection. The robot was manually positioned at each of those points to sense the surrounding environment and the robot estimated the homing directions, using the suggested snapshot model algorithm.

### 2.2. Depth Information Processing

**Figure 2:**
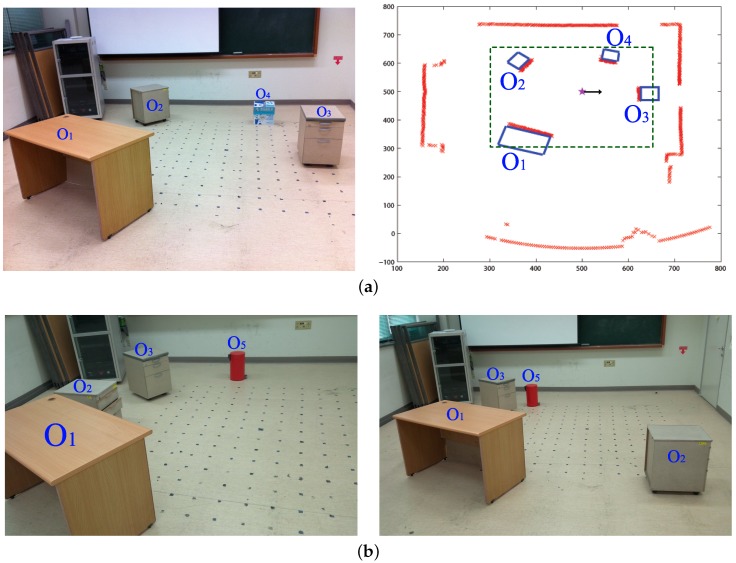
Test environments (**a**) environment env1 (**left**: environment, **right**: distance map at the home location with a laser sensor, *: home location); blue lines: top-view of objects, green dashed rectangle: interest zone for test points, called Region 2, black arrow: the reference direction (zero degree). The laser sensor collects the range data in a counterclockwise direction, and red *x* marks indicate detection points read by the laser sensor; (**b**) environments, env2 (**left**) and env3 (**right**) ($O_{1},\ldots ,O_{5}$: objects).

**Figure 3:**
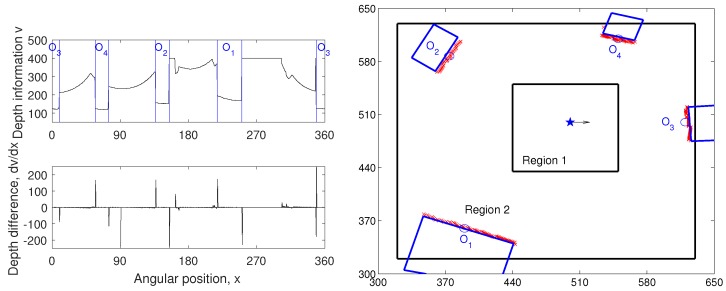
Example of depth snapshot (**left**: depth snapshot for env1 and the derivative of the depth, **right**: close landmark objects (blue circles with labels) extracted from the depth snapshot). Black rectangles with label ‘Region 1’ and ‘Region 2’ (green dashed rectangle in Figure 2) include testing points.

The experimental environment is a 3 m × 3 m area containing objects such as a desk, drawer, and trash bin. The panoramic depth information obtained with a mobile robot is shown in Figure 2. By using the depth information for a certain height of the view, we obtain a one-dimensional depth snapshot. Only this one-dimensional snapshot will be used as the environmental information for our navigation experiments. Figure 3 shows examples of the depth snapshot, which has 720-pixel width with a resolution of 0.5${}^{\circ }$. Since we only sample distance information at a certain height, it may not represent all of the information about every landmark in the environment. However, we assume that it can give sufficient information about the landmarks in terms of the mobile robot’s view, especially for our navigation experiments.

In our experiment, the DELV method [21] will be tested for landmark-based navigation. In order to use the DELV method, certain ‘landmarks’ should be selected. A set of landmarks can be obtained from segmenting the depth information. This pre-processing for a set of landmarks has been tested with vision [21]. Using visual information, objects can be found with image pixels based on similar color and the brightness, that is, by a feature extraction algorithm with clustering. Using the depth information, two main criteria were used for the landmark extraction. The first criterion is based on the difference of the depth values. The differences of the distance value between neighboring points in the panoramic snapshot are calculated. Since the boundaries of the landmarks would have large variations in their depth values, we set points with large difference as the preliminary landmark boundaries. Assuming continuity of the depth values in each landmark, candidate areas for the landmarks are selected. The second criterion is the threshold value of the depth information. Within the preliminary landmark area set by the first criterion, several regions with small depth values (small distances) are selected as landmarks.

An example of depth snapshot and the corresponding landmark extraction results are shown in Figure 3. Left figures of Figure 3 show the depth values for the $360^{\circ }$ surroundings of a mobile robot at the home location and its derivatives (for edge detection). The candidates were selected based on the points with large depth derivatives. Comparing the position of the selected landmarks and the depth information, we can observe large variations at the boundaries of the landmarks and comparatively small variations inside the landmark areas. The objects closer to the agent were selected prior to the background areas, using the thresholding value in the second criterion. If the location is closer to the wall than to the other objects, the agent could regard the wall area as one of the landmarks.

### 2.3. Navigation Algorithm: DELV

**Figure 4:**
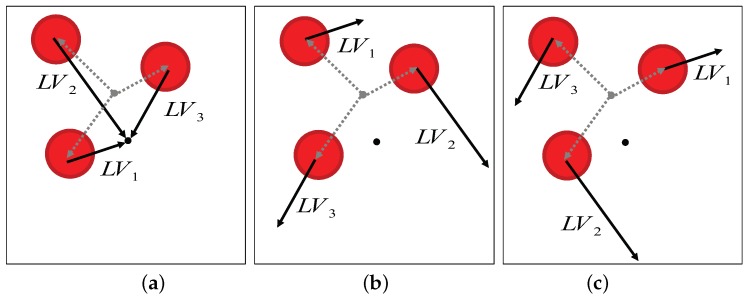
Projecting landmark vectors onto the reference map in different arrangement orders, and the correct order can be determined with the deviation of end points of projected landmarks ((**a**) for the best matching case, and (**b**,**c**) for other matching cases).

For the snapshot navigation, we initially tested the Distance-Estimated Landmark Vector (DELV) model [21], which is an efficient landmark-based homing navigation algorithm. Each landmark vector in the DELV method has information about an angular position of the landmark, given as the angle of the vector, with the distance to the landmark as its length. The landmark vectors obtained at the home location create a reference map. The set of landmark vectors at an arbitrary location is projected on the reference map with an appropriate arrangement order and direction (see Figure 4). The landmark vector order and the heading direction are estimated by rotational projection of vectors. When the landmark vectors are appropriately matched with the corresponding ones in the reference map, that is, with the right order of landmark vectors, the end points of the projected landmark vectors would converge to the current location. In other words, we can find the appropriate landmark arrangement order by monitoring the deviation of the projected end points. The arrangement order with the minimum deviation of end points would indicate the appropriate matching. In the same way, we can project the landmark vectors by rotating the heading direction and estimate the current heading direction in the reference map, if there is no reference compass available. Ultimately, the above landmark arrangement process determines the homing direction. Here, we suggest two new methods, C-DELV and H-DELV that have different landmark vectors from the depth snapshot.

### 2.4. Navigation Algorithm: C-DELV Method

The DELV method collects landmarks by assigning one representative landmark vector for each landmark area or segmented region. The centroid of the angular positions of points in the landmark area and the average of the depths of the points are the angle and the distance for a landmark vector. In this way, we obtain one landmark vector for each selected landmark area. For example, in Figure 3a, there would be four landmark vectors at a given position. Here, the size of landmark areas is not considered for the landmark representations.

Now, we consider another type of landmark representation from a depth snapshot. The landmark representation defines every point in the landmark area as the individual landmark vector, but the background area is ignored. Only dominant landmarks near the robot are collected, and we will use this set of landmark vectors for homing navigation. We call it the C-DELV method (with cloud-like landmark vectors). Since the landmark areas are composed of several points of similar but slightly different distances, each point is considered as a landmark vector in this representation of landmarks and a set of landmark vectors point to a segmented object.

**Figure 5:**
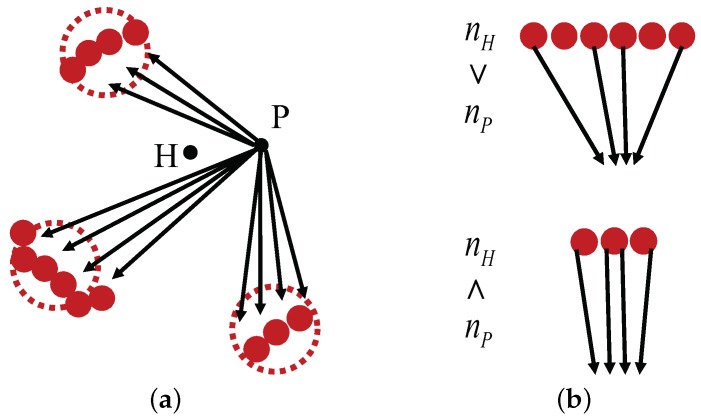
Landmark vector matching in the C-DELV method (**a**) landmark vectors at a position *P*; (**b**) vector matching example with $n_{p}=4$ (top: $n_{h}>n_{p}$, bottom: $n_{h}<n_{p}$).

In Figure 5a, large dotted circles indicate selected landmark areas while small red circles indicate the landmark vector points that belong to a selected landmark area from the home location *H*, according to the C-DELV method. Therefore, the reference map is given by the red circles in the figure area. Black arrows are the perceived landmark vectors at the current new location *P*. In rotational landmark vector matching, each vector set should be projected appropriately on the reference map. However, in the rotation, the number of landmark vectors for the landmark area may not be the same as that of the same landmark area in the reference map. As shown in Figure 5b, the number of landmark vectors included in the same area, for example, $n_{H}$ and $n_{P}$, can vary depending on the viewing position *H* and *P*. Therefore, the matching procedure was modified to appropriately distribute the landmark vectors to the reference map as shown in Figure 5b. In this way, each landmark area yields its projected end points to estimate the current location. Large-sized landmark features contribute more to estimates of the current position. When projecting one perceived landmark area to the one in the reference map, different numbers of landmark vectors should be matched. An example of the diagram is shown in Figure 5.

### 2.5. Navigation Algorithm: H-DELV Method

For another representation of landmarks, we can consider all distance points or sampled points from the omni-directional depth snapshot as landmarks. The distance points cover background areas as well as landmark areas. We call it the H-DELV method (with a holistic view of environment landscape). The method simply uses all the points or a set of uniformly sampled points from the depth snapshot. The panoramic depth snapshot from a laser sensor has 0.5 degree resolution and a total of 720 pixel depth information for the omnidirectional view. By sampling the panoramic depth information at preset angle intervals, the number of depth measurements is reduced, but we still maintain the same overall information about the environment. For example, if we sample the depth panorama at $4^{\circ }$ intervals, a set of distance information of the environment with 90 samples is obtained. The whole set of distances are represented as landmark vectors. This procedure is a holistic approach without any landmark segmentation procedure. Unlike the image-warping method [14], the H-DELV method estimates the current position with a collection of landmark distances by calculating the relative distance from the home location.

### 2.6. Characteristics of the Navigation Algorithms and Weighted Landmark Vectors

The above methods, the DELV, C-DELV and H-DELV methods, have a difference in terms of how they choose landmarks or landmark features from a collection of depth information. Any possible approaches can be available for the extraction of landmark features. Here, the DELV method includes a clustering process using a set of depth information, but removes the background area. The C-DELV method is similar to the DELV, but it uses each depth point as a landmark. The H-DELV method considers all of the depth information, including the background. We will compare the DELV and the C-DELV to see if the clustering process from the depth image is more effective. The comparison of the C-DELV and the H-DELV will check whether or not the background depth information improves the homing performance.

In the original DELV method, the end points of the projected vectors were averaged to estimate the current location. However, as we use the depth sensor to obtain the landmarks in the environment, its accuracy depends on the measuring distance. It can be assumed that the farther the landmark is, the lower the accuracy of the estimated distance becomes. Based on this assumption, the end points of the projected vectors in the reference map can be weighted differently when calculating the current location. Instead of averaging each projected point equally, the points from the shorter landmark vector are weighted more than the others. The weight for each point was computed as the inverse of the length of the landmark vectors. Therefore, the projected points from closer objects are considered to be more important than those from farther objects. By weighting the end points in the current position estimation, the position estimation may be expected to be more accurate. We apply the weighted landmark vectors to the above three methods.

## 3. Experimental Results

The three different methods described above are applied to the omni-directional depth information. We use the collected landmarks in the navigation method and show a homing vector for each location. In order to show the performance of the method, we took panoramic depth snapshots at many locations in the environment. Figure 2 shows the experimental environment for the depth image. The size of the environment is about 3 m × 3 m with objects such as a desk, a chair, drawers, and a trash bin. We chose a set of locations across Regions 1 and 2 from the regular grid pattern on the floor of the environment (see Figure 1) that were not too close to any of the objects in the three environments (see Figure 2). Eleven test points were located within Region 1 and 31 points were located within Region 2. Vector maps show the homing vector determined for each point. The performance of each method is evaluated using the angular error of the homing vector and the success rate of homing from each starting point. We test each method both with and without the reference compass to see if the suggested method can estimate the homing direction effectively even without the reference compass, but only depending on the depth snapshot. In addition, we test the three methods, DELV, C-DELV and H-DELV, using the distribution of weights as the inverse of the distances over landmark vectors.

### 3.1. The DELV Method

The DELV method has landmark vectors composed of representative centroids of selected landmark areas. It requires segmentation of the landmark areas and then one landmark vector is assigned for each area. The corresponding landmark vector for each area is composed of the centroid angle of the points included in the area, and the average distance of the points.

**Figure 6:**
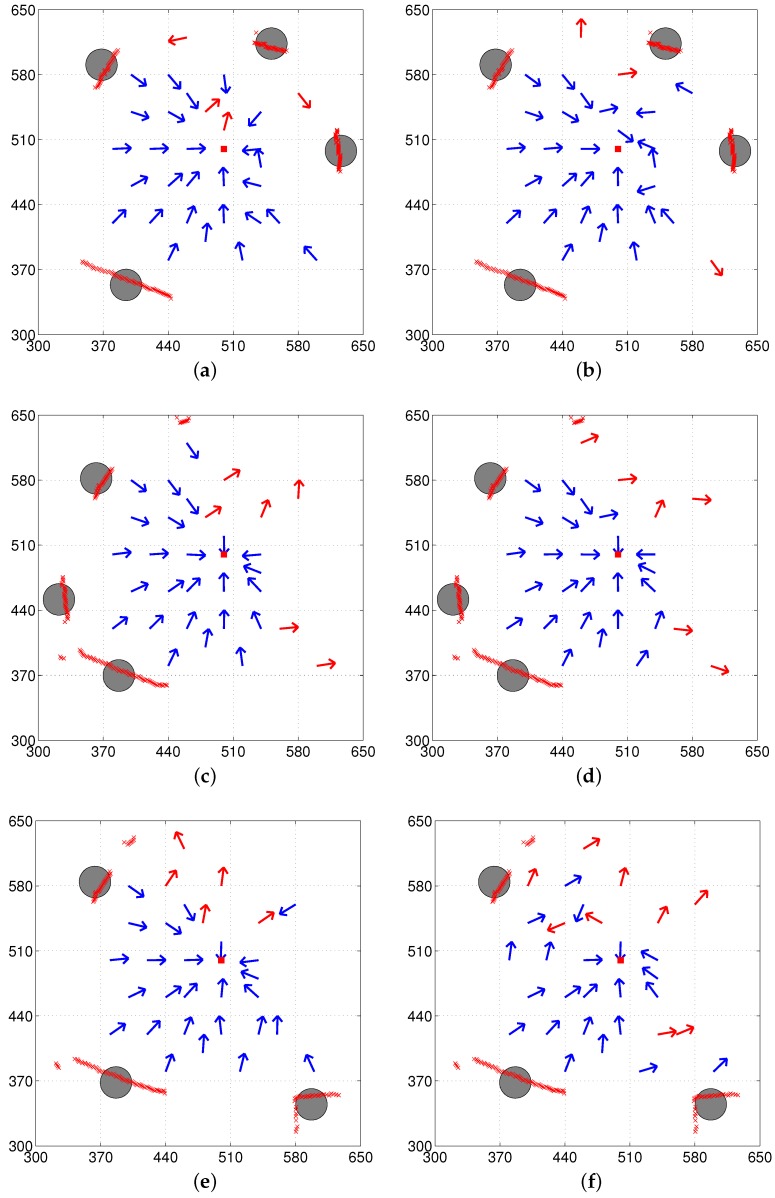
Vector map results with the DELV method (from the omni-directional depth snapshot, left column: with reference compass, right column: without reference compass); (**a**,**b**) env1; (**c**,**d**) env2; (**e**,**f**) env3 (blue and red arrows indicate small and large homing errors, respectively, and circles represent close landmark objects obtained after segmentation).

The circles in the vector maps shown in Figure 6 indicate selected landmark vectors at the home location, which is then stored as the reference map. The center of each circle indicates the end point of the actual landmark vector from home and the radius of the circle is arbitrarily set for the graphical representation. At each location, the landmarks were extracted and represented as the corresponding landmark vectors in order to estimate the homing vector. The direction of the arrows in the vector map indicates the decided homing direction at each point while the red square at (500, 500) indicates the home location. Blue and red arrows in the vector maps indicate angular errors with a positive sign to return home [−90${}^{\circ }$, 90${}^{\circ }$] and errors with a negative sign (greater than $90^{\circ }$), respectively.

**Figure 7:**
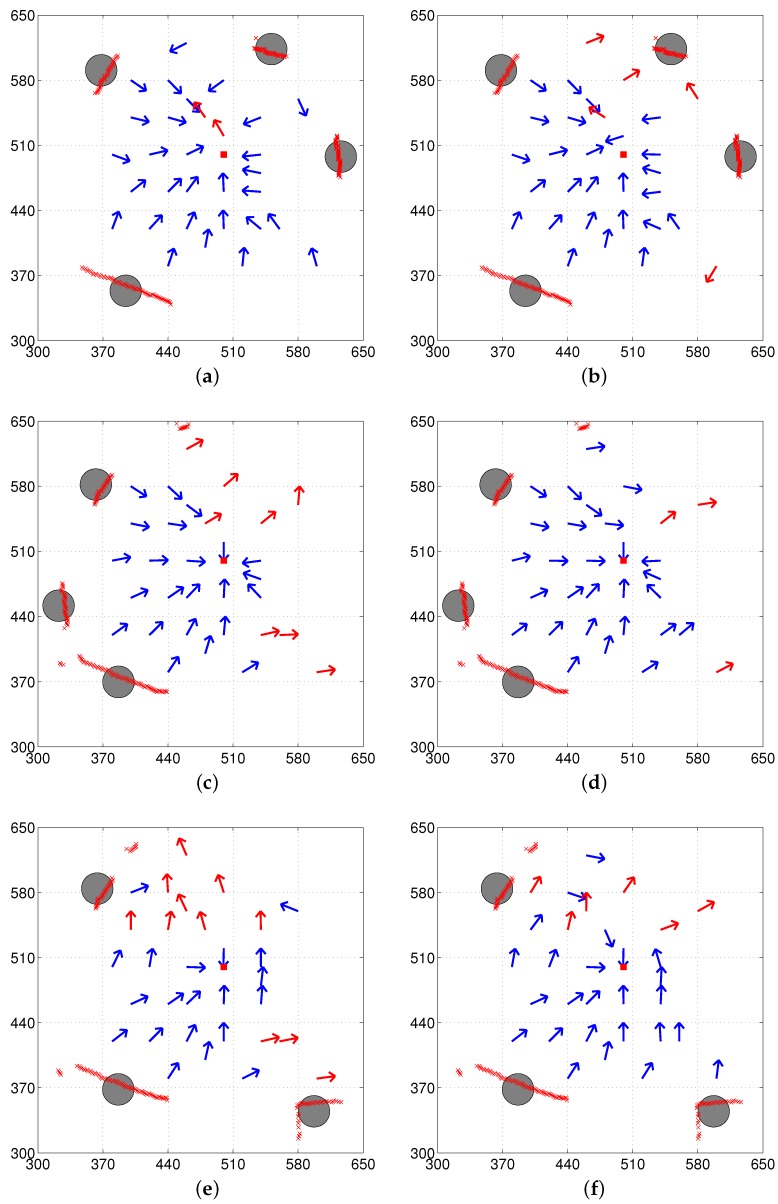
Vector map results with the DELV method but weighted landmark vectors applied for the position estimation (left column: with the reference compass, right column: without the reference compass); (**a**,**b**) env1; (**c**,**d**) env2; (**e**,**f**) env3.

The vector map in Figure 6 shows the results of homing navigation using the DELV method. Figure 6a,c,e show the results with the reference compass while Figure 6b,d,f show the result without the reference compass. The homing navigation algorithm requires estimating the heading direction and the arrangement order among all possible heading directions and arrangement orders. With the reference compass, the method can reduce the computational cost to estimate the current heading direction using the distance map, and one task of finding the appropriate landmark arrangement order still remains. The vector maps shown in Figure 7 show the results with the weighted landmark vectors. It has similar results compared to those in Figure 6. The homing performance greatly depends on the environment, that is, the landmark distribution. They show smaller angular errors in homing vectors, which may lead to higher success rates of homing (more detailed analysis will be described later)

In fact, there is no significant difference between the DELV method and its weighted vector method as shown in the vector map results. The DELV method may lose some landmarks in an environment in the feature extraction process. If a landmark area is relatively small (i.e., the angular size is smaller than 5${}^{\circ }$), it is filtered out. If the landmark area has a distance larger than 550 cm, it is regarded as a wall, and it is not selected as a landmark.

### 3.2. The C-DELV Method

The C-DELV method is similar to the DELV method, but it considers points belonging to the selected landmark area as landmarks. After segmentating landmarks based on the omni-directional depth information, all the points included in the landmark area are represented as landmark vectors. The background area, which is not the landmark area, will not be considered as landmarks. While only one landmark vector was assigned for each landmark area in the DELV method, the C-DELV method has several landmark vectors in a single landmark area. Therefore, the matching process of landmark vectors to the reference map also needs to be modified. In the C-DELV method, each landmark area is rotated and projected onto the reference map.

**Figure 8:**
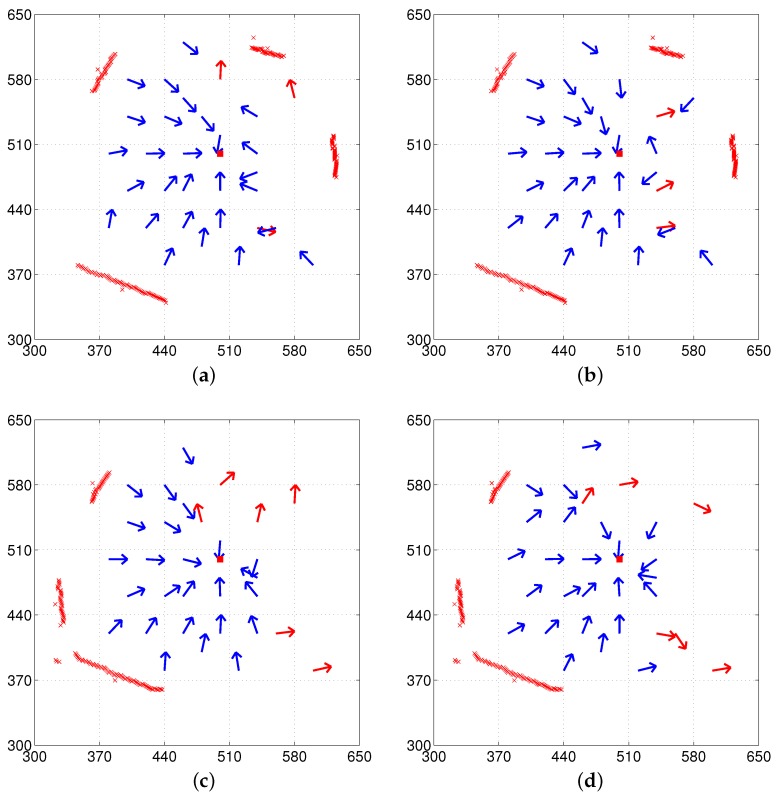

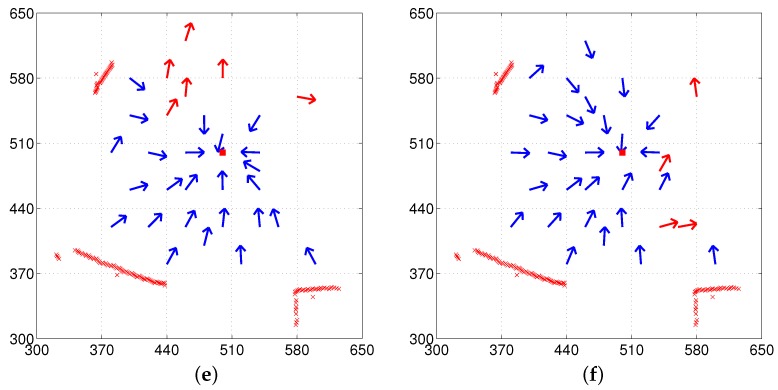
Vector map results with the C-DELV method (left column: with the reference compass, right column: without the reference compass); (**a**,**b**) env1; (**c**,**d**) env2; (**e**,**f**) env3 (x marks indicate depth sensor readings before segmentation of depth snapshot).

We tested the C-DELV method in the same environments: env1, env2, and env3. The vector map results with the C-DELV method are shown in Figure 8. The points marked with ‘x’ are the landmark vectors stored in the reference map that correspond to small red circles in Figure 5a. From the figures, we observe that the C-DELV with a reference compass is often better than the DELV method. Better homing directions are observed at many test positions. With the C-DELV, the weighted landmark vectors have no positive effect on the performance, but rather decrease the performance (not shown here). Detailed evaluations will be given in the Performance Evaluation section with the angular homing errors and the homing rate.

### 3.3. The H-DELV Method

In the H-DELV method, all distance points or same points including the background areas from the omni-directional depth snapshot are considered as landmark vectors. This type of landmark selection does not require the segmentation process needed in the DELV method. Every sampled point is considered as a landmark vector. Once we fix the number of samples for the landmark vectors, the same number of landmarks is taken in the reference map as well as at other locations. However, the computing time for estimating the homing vector is inversely proportional to the number of samples in the depth snapshot. An appropriate number of samples would be desirable, since snapshots that are too sparsely sampled may not reflect the environment closely.

**Figure 9:**
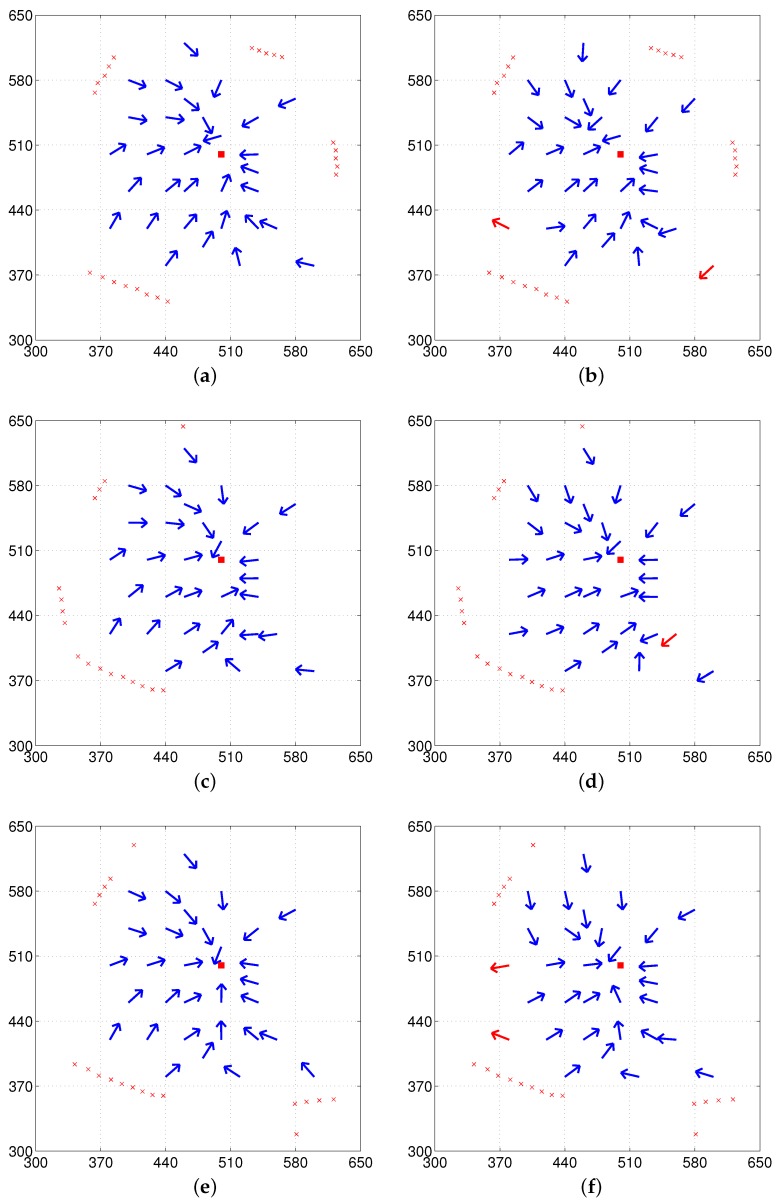
Vector map results with the H-DELV method (left column: with the reference compass, right column: without the reference compass); (**a**,**b**) env1; (**c**,**d**) env2; (**e**,**f**) env3 (red x marks indicate landmarks for H-DELV, which uses a uniform distribution of depth samples including far background areas not shown, although they are used.

We tested again the H-DELV method in the same environments. Figure 9 shows the vector map results with the H-DELV method. The points marked with an ‘x’ in Figure 9 are the 90 landmarks stored in the reference map, which were sampled at $4^{\circ }$ intervals in the panoramic depth snapshot. The red square indicates the home location and the arrows show the decided homing vectors at each point with the given algorithm. Since this method uses 90 landmark vectors, the landmark arrangement matching is iterated 90 times.

It seems that weighted position estimation does not largely affect the result with the H-DELV method, since the results with the H-DELV method are similar to those with the weighted landmark vectors (not shown here). More detailed evaluation will be given in the following subsection along with the angular error of the homing vectors and the success rate of homing at each location.

### 3.4. Performance Evaluation

For each task over three different environments, we can evaluate the homing performance based on the angular error of the decided homing vectors and evaluate the success rate of homing based on the homing vector sequence. The angular errors of the homing vectors are defined as the difference between the decided homing direction and the ideal homing direction. From each point on a mobile robot, we can draw a straight line to the home location, and the angular difference between the line and the homing vector from the point is considered to be the angular error. The performance of the homing navigation algorithm may vary depending on the distance from the home location. We thus assess the angular error by dividing the environment into two regions. Region 1 is the region close to home location, and Region 2 is the zone that surrounds the landmark but is farther from home than Region 1. The two regions for each environment are shown in Figure 3. Outside Region 2, some landmarks may be occluded, and this degrades the homing performance (not shown here).

#### 3.4.1. Performance with Angular Errors

**Figure 10:**
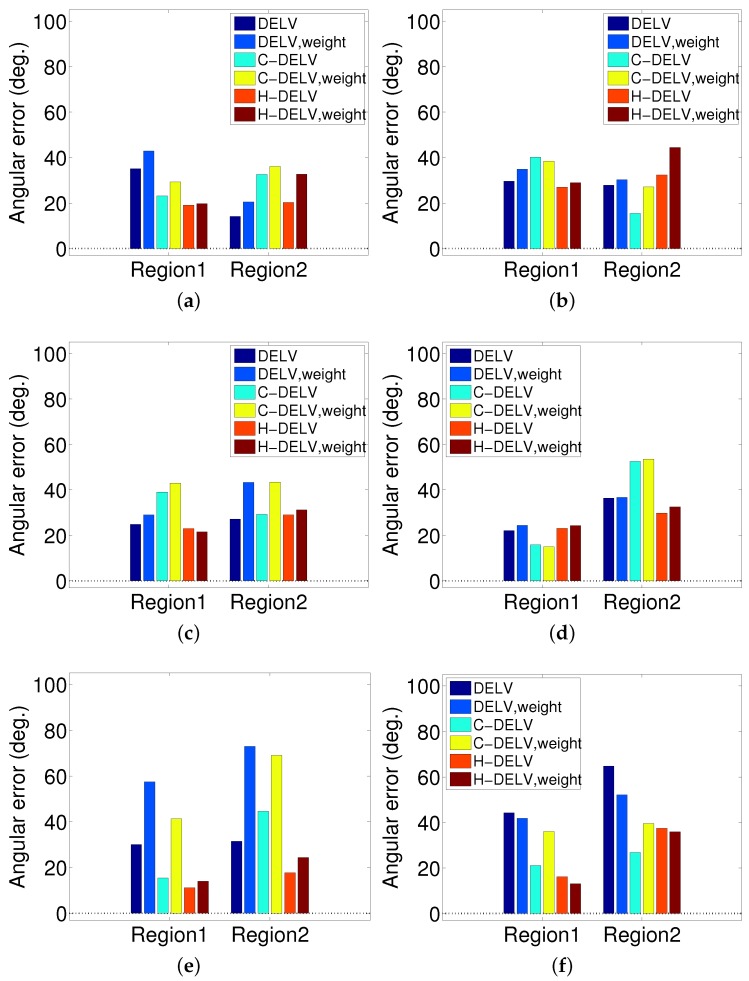
Angular errors for three different methods in Regions 1 and 2 (left column: with the reference compass, right column: without the reference compass); (**a**,**b**) env1; (**c**,**d**) env2; (**e**,**f**) env3.

The angular errors for each method are displayed in Figure 10. Different landmark types (weighted or not weighted) and their homing method are applied to three environments shown in Figure 2. For each method, the angular errors for points in each region were averaged and plotted as a bar graph. In most of the methods, the angular error increases as the robot gets further from the home location, and they show slightly larger angular errors without a reference compass than with a reference compass. Generally, even without a reference compass, each method can find homing vectors effectively under a reasonable limit. This indicates that the depth snapshot image alone may be sufficient to guide the homing navigation. The H-DELV method mostly shows smallest angular errors in Region 1 (near the home location). In addition, even without a reference compass, the H-DELV method shows comparatively steady performance. When the robot is far from home location, the performances of the three methods vary depending on the environment.

In Figure 6, Figure 7, Figure 8 and Figure 9, each method has many test points, leading to reasonable homing directions, but there are a small number of points with incorrect homing directions. Those points significantly affects the averaged angular error performance. For example, Figure 6a has an angular error of almost 180 degrees near the point (510, 520). Thus, we counted the number of robot positions with relatively small angular errors, that is, within the range of [$-45^{\circ }$, $45^{\circ }$] in angular errors, and the rates of those desired angular errors were shown in Table 1. We can see that the H-DELV method shows the best performance in terms of the angular errors at many test points in the three environments (see the best results in bold fonts in Table 1). The DELV and C-DELV methods have some points with large angular errors, which degrades the performance in terms of the averaged angular errors.

The angular errors greatly depend on a set of landmarks that have been selected from the depth image. At some test points, the homing directions were estimated incorectly. So far, we have tested the methods in a restricted zone near the home position. If the robot moves far away from the home position, the landmarks in the environment can be changed, and the landmark matching process may not be effective. Similar results can be expected in some environments with occlusions.

#### 3.4.2. Performance with Homing Success Rate

**Figure 11:**
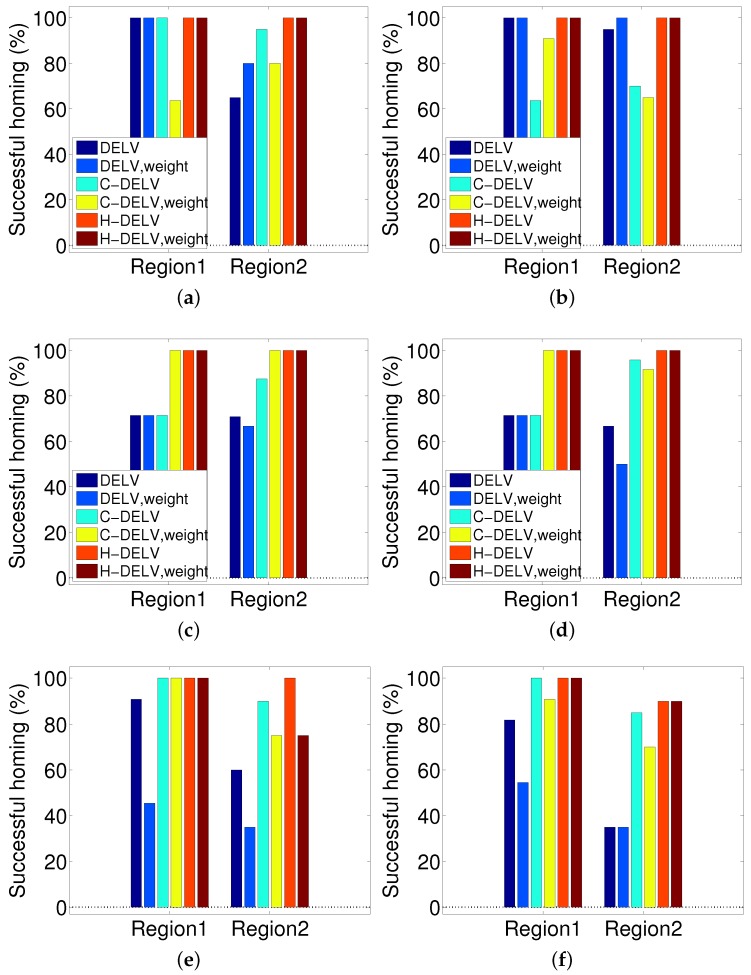
Success rate of homing for three different methods in Regions 1 and 2 (**left**: with the reference compass, **right**: without the reference compass); (**a**,**b**) env1; (**c**,**d**) env2; (**e**,**f**) env3.

The success rate is the ratio of starting points that could finally reach the target location. That is, it corresponds to the percentage of the catchment area to the goal position. We calculate the distance error between the current position and the home position, and if the distance is smaller than a threshold (10 cm), then we take the trial as a success. If the robot does not reach the target zone in less than 30 s, it is counted as a failure. As with the angular error assessment, the area is divided into two regions and the homing rate is evaluated for each region. The results are shown in Figure 11. The angular error increases as the distance from home increases. As a result, the success rate decreases as the robot is farther away from home location. Interestingly, the H-DELV method shows the highest success rate in homing. The DELV and C-DELV methods do not show good performance in homing even though they have similar level of angular errors in Figure 10. This result is due to a single trap point near home. For example, in Figure 7b, the homing vectors collide at the point (475, 550). This one trap point leads to the most of the failing cases in the methods. The success rate alone cannot represent the performance of the method sufficiently and therefore we show two other types of evaluations, the vector maps and angular errors.

Among those three methods, a holistic approach, that is, the H-DELV method was the most effective in the performance evaluation. The DELV method needs a feature extraction process in which the landmarks are identified from the depth information. The C-DELV method also needs a process of disciminating landmarks from the background. At some robot positions, landmarks that are found at the home location may not be selected in the filtering process, which significantly affects the performance of the landmark arrangement process. In addition, the number of landmarks may be changed depending on the position of the robot or a different set of landmarks may be selected from the depth image. In contrast, the H-DELV method uses more information regarding the background distances. A holistic view of the whole omnidirectional information regards all of the depth information as a set of landmarks with a landmark for each sensor reading, which is why the H-DELV method is more powerful and more robust than the DELV and C-DELV methods.

#### 3.4.3. Missing or Added Objects

**Figure 12:**
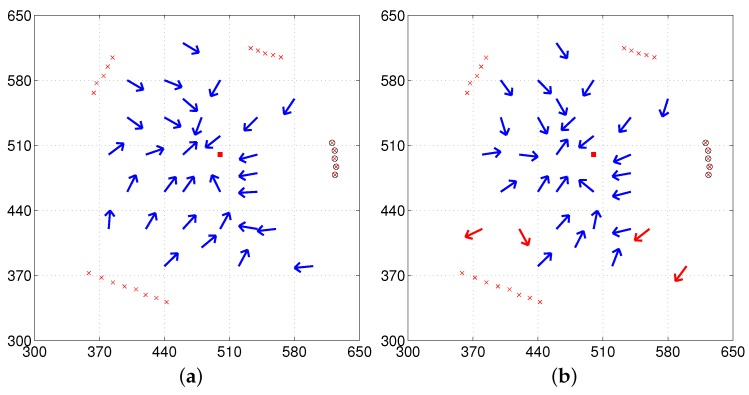

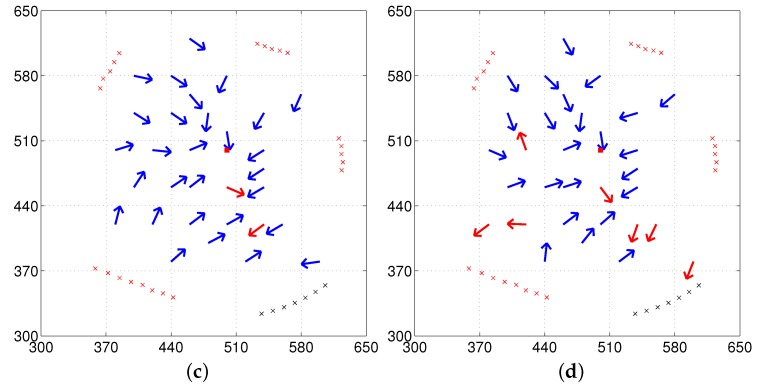
Experiments with missing landmarks or added landmarks (left column: with the reference compass, right column: without the reference compass) (**a**,**b**) env1 with an object removed at home location and (**c**,**d**) env1 with an object added at home location; black circles (at the right side) in (**a**,**b**) indicate landmarks removed only at the home position and black x marks (at the lower right corner) in (**c**,**d**) indicate landmarks added only at the home position (red x marks represent landmarks observed at any position except the home position and far background areas not shown, although they are used).

In the above experiments, we assume an isotropic landmark distribution where most of landmarks available at the home location are observed at other locations, although the viewing direction tends to change the landmarks and the size of landmark objects. If a robot is positioned outside the Regions 1 and 2, the robot sees very different landscapes and the snapshot model cannot be applied, since it violates the isotropic condition. Environmental changes, such as a new object added or one of objects missed, severely influence the landmark distribution. Here, we consider that kind of change in env1 as shown in Figure 12. Figure 12a,b show the situation where a new object is added (three large objects observed at home location, but four large objects during testing) and partial occlusion occurs. The homing performance becomes worse, as expected, than in the isotropic environment shown in Figure 9. Especially at positions far from the home, large homing errors are found. Figure 12c,d show the situation where an object is missing (five large objects observed at home location, but four objects during testing). Large homing errors are observed at positions close to the missing object. Generally, the homing performance with the reference compass is much better than that without the reference compass in both situations.

The homing errors become large when landmarks change in the environment, but robots can return home at many positions and the suggested method has a potential to return home without further sophisticated algorithm. More knowledge about the environment or landmarks could possibly improve the performance rather than only memorizing a snapshot at a target position. In many non-isotropic environments, the snapshot model may experience landmark mismatch at a pair of locations, which degrade the homing performance. A holistic view of landmarks covering the background area has a higher probability to compensate for missing or added objects. Interestingly, some insects show homing errors when the landmark distribution is changed or landmarks disappear [11,19]. The snapshot model is still valid among biological animals.

#### 3.4.4. Resolution of Samples in the H-DELV Method

**Figure 13:**
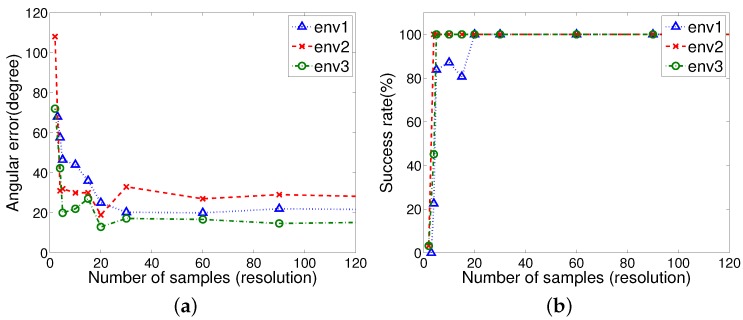
Performance vs. resolution in the H-DELV method (**a**) angular error vs. resolution; (**b**) success rate vs. resolution.

Figure 13 shows that with the H-DELV method the performance depends on the resolution of the distance sensor readings. Interestingly, once the number of samples is greater than 30, then the performance is not much improved in terms of success rate by additional samples. Instead, a relatively reasonable number of samples to cover the environmental characteristics is sufficient for achieving good homing performance. We also tested the entire set of 720 omnidirectional distance readings (0.5 degree resolution), and the performance result is only a little different from that with 90 samples (4 degree resolution). Even only 20 samples (18 degree resolution) provide a perfect homing rate for all the three environments. Uniformly distributed samples including the background area affect the homing performance, when we compare the DELV or C-DELV with the H-DELV method. From the result, we infer that a global view of the environment with a holistic approach can be useful to handle real environment problems.

## 4. Discussion

We tested three landmark-based methods with the omnidirectional depth image. The difference of the methods depends on how to extract candidate landmarks from the depth image. The original DELV (distance-estimated landmark vector) method as a landmark-based method is based on feature extraction over visual images. The DELV method chooses a small number of landmarks based on a clustering process, and the C-DELV method selects objects near a given robot position, and regards the depth pixels of those objects as landmarks. The H-DELV method uses a holistic view of the omnidirectional depth information, including the background depth. As a result, the H-DELV method takes a given environment as an isotropic environment by collecting all of the surrounding depth information. In the isotropic environment, the suggested H-DELV approach is very effective. If the background depth is not available and only isolated landmarks are available, then the H-DELV method becomes similar to the C-DELV method. From Table 1, it is difficult to say whether or not the DELV method is better than the C-DELV method. Their homing performance varies depending on the environment.

If there are measurement errors with the depth information, it may degrade the performance. The DELV and C-DELV methods are more heavily influenced by the errors. Some missing landmarks or no mismatched landmarks degrade the performance. In contrast, the H-DELV method uses uniformly distributed samples over the omnidirectional depth information, and some small errors in a part of samples do not influence the homing performance significantly. According to the DELV method [21], there is a global convergence of the homing vector to reach the home location. That is, an agent can ultimately reach the home location, if the same landmarks are observed. Some trap points can be found with measurement errors, especially in the DELV and the C-DELV method. To handle this problem, a holistic approach over landmarks is suggested in this paper. The suggested method has no need to consider the shape, size, and density of obstacles, and only the depth information from a mobile agent is sufficient to estimate the homing directions. However, visual navigation may be influenced by the factors of the shape or size of obstacles.

### 4.1. Resolution of Samples

The DELV and C-DELV methods have a feature extraction process over *n* samples in the one-dimensional depth image, and an arrangement matching process over *m* landmarks, if *m* landmark features are selected from *n* samples. Then, the computational complexity can be represented as $O(n+m^{2})$. The H-DELV method has time complexity $O(n^{2})$, since it has no feature extraction process. The number of samples in the H-DELV method can be reduced to 30 as Figure 13 shows resolutions not affecting the performance. As a result, the above methods take a little time to determine the homing direction.

### 4.2. Comparison with SLAM

Our experiments show that the laser sensor information is more powerful than a vision sensor. The laser sensor has a high cost compared to a vision camera. Many SLAM methods use laser sensors with high accuracy. The methods focus on localization of a mobile robot in a complex cluttered environment. Most of the researchers use Kalman filtering of laser sensor readings to obtain highly accurate localization information. Actually, it requires the computationally intensive process of probability estimation. Our approach has a goal of reaching the home position at an arbitrary position. The method shows that the robot can return home without localizing the current position and the home position in a given environment. The algorithm simply compares the landmark vectors and determines the homing direction in a short time, without accumulating the map information of the environment in memory. This is an advantage of the suggested approach for homing navigation. In a complex cluttered environment including many landmarks or objects, the suggested approach may need more computation using the landmark vectors for an accurate homing direction.

### 4.3. Connection with Bio-Inspired Model

The above homing navigation approaches follow the snapshot model, which compares the two snapshot images at a pair of positions. The model assumes that the majority of landmarks are observed commonly to guide the navigation and the environment is almost isotropic. Complex cluttered environments with many occlusion points no longer follow the assumption, and the method would need several waypoints as a set of target positions [30]. For each waypoint, local homing navigation can then be applied. A series of waypoint searches may ultimately lead to the final goal position.

As mentioned in the Introduction section, bio-inspired homing navigation methods can be roughly divided into two categories: holistic methods such as the warping and DID methods, and landmark-based methods such as the ALV and DELV methods. The holistic method uses the information in the snapshot as a whole, and does not require any pre-processing on the snapshot information whether it is from vision or depth sensors. However, the landmark-based navigation methods require a collection of selected landmark information from the snapshot, and therefore the performance heavily relies on the landmark extraction. The landmark-based methods, if landmarks are correctly identified and matched, usually provide much better homing results than the holistic methods, but performance degradation may happen in real environments, due to errors in landmark identification and matching. The H-DELV method as a holistic approach actually possesses nearly all the environment information, including the background area. There would be some mismatch points between depth snapshots, but the majority of samples point to the same landmark area, which can reduce the homing error.

The holistic approach is more useful in a static environment that has no symmetries, but has a sufficient number of landmarks. If a set of distance vectors are all regarded as landmarks, more landmarks would be helpful to guide homing. Omnidirectional depth information can lead to more successful homing. That is, in the environment where landmarks are distributed over the whole angle directions, better estimation of homing direction is possible. If landmarks on one side are observed and other landmarks are too far (out of the sensor range), the environment may experience relatively large homing errors.

We note that ants can memorize key objects in their visual field using features of the skyline, for example, tree tops as a navigation guide, and, on later trips, they can compare the snapshot memories with the currently observed view [31,32,33,34]. That is, they can estimate their distance from landmarks by the change of the skyline. Inspired by the snapshot model with distance information, we explored a homing navigation strategy based on the distance of landmarks. We argue that the holistic model suggested above might resemble the snapshot model observed in ants. Even a low resolution of snapshots are sufficient to guide the homing navigation. Our model with various angular resolutions ranging from 0.5 degree to 10 degrees showed a perfect homing rate.

## 5. Conclusions

In this paper, we tested homing navigation based on the snapshot model in real environments, where the mobile robot is positioned at an arbitrary position in isotropic-like environments and is supposed to return home. Here, two new methods, C-DELV and H-DELV, were applied to a homing navigation model and compared with the DELV method. In addition, we tested weighted landmark vectors in the homing navigation approach, where objects that are closer to a given position have larger weights for estimating the current position. To evaluate the performance, we measured the angular error of the homing direction and the success rate of the homing.

The H-DELV algorithm, a holistic approach with the omnidirectional depth information, showed the smallest angular error and highest success rate. Even a low resolution of depth samples covering the background estimate the homing directions more accurately. With the H-DELV method, the reference compass is greatly helpful for reducing homing errors. We argue that a holistic view of landmark distances without landmark segmentation process provides better homing performance than a landmark distribution processed with the feature extraction process. In natural landscapes of the environment, the holistic approach can lead to robust homing navigation. The above results may indirectly support the hypothesis that the insect navigation might use the snapshot model with a holistic view of distance vectors [31,32]. Interestingly, the results show that a new order (weight) of landmark vectors does not have much impact on the homing performance.

Our homing navigation method can be applied to cleaning robots or exploration robots in industry that need a battery recharging system or that have a goal to move towards a target position. For successful homing navigation in large areas with many objects or in a complex non-isotropic environment with many occlusions, the suggested method needs several waypoints like homing positions. Robot homing toward a sequence of waypoints may guide the agent to the home position. We leave that study as a future work.

## Figures and Tables

**Table 1:** Percentage of test points with small angular errors (errors are in the range of [−45${}^{\circ }$, 45${}^{\circ }$] and (w) indicates the method with weighted landmark vectors).

Unit : %		DELV	DELV (w)	C-DELV	C-DELV (w)	H-DELV	H-DELV (w)
Env1	with compass	83.9	83.9	77.4	80.7	**96.8**	80.7
	no compass	74.2	77.4	80.7	77.4	**80.7**	74.2
Env2	with compass	80.7	71.0	77.4	67.7	**90.3**	80.7
	no compass	74.2	71.0	67.7	67.7	**80.7**	74.2
Env3	with compass	83.9	38.7	77.4	51.6	**96.8**	**96.8**
	no compass	48.4	54.8	80.7	61.3	**87.1**	**87.1**
